# Modelling Stakeholder Valuation: An Example Using the Surgical Treatments for Gastroesophageal Reflux Disease

**DOI:** 10.7759/cureus.19559

**Published:** 2021-11-14

**Authors:** Qizhi V Zheng, Vic Velanovich

**Affiliations:** 1 Surgery, University of South Florida, Tampa, USA

**Keywords:** total incisionless fundoplication, antireflux surgery, gastroesophageal reflux disease, stakeholder perspective, treatment value

## Abstract

Background

Assessing the value of a treatment is of great importance. Typical methods are directed toward policy decisions. However, individual stakeholders will have different valuation based on their interests.

Methods

Formulas were developed to quantify the value of a treatment from the patient, surgeon, hospital, and private third-party payer. These formulas are based on observed factors that go into treatment decision-making for each stakeholder. Using the example of four surgical treatment options for gastroesophageal reflux disease, values for each factor were obtained from publically available documents or were arbitrarily estimated.

Results

From the patient perspective, the laparoscopic Nissen fundoplication (LNF) provided the best value at 2.99 quality-adjusted life years per $1,000 spent. From the surgeon perspective, it provided the best value at $752.20 earned per hour effort. From the hospital perspective, LNP provided the best value at $3,446 earned per episode of care. Lastly, from the third-party payer perspective, total incisionless fundoplication provided the best value at $13,336 per year.

Conclusions

Because value is measured differently for each stakeholder, there will be conflicts as to how treatment options are valued.

## Introduction

There has been a great deal of attention paid to assessing “value” in health care. These have generally relied on some variation of the following equation: value = quality/cost. The notion here is that the higher quality of care delivered at a lower cost, the higher the value. This idea has several difficulties. Firstly, as cost goes to 0, value will be high as long as quality is some value >0. Prima facie, this is not an acceptable proposition as most patients would not accept very poor quality because it is very cheap. Most individuals would, if having the means, willingly pay more for better quality. The second issue is how “quality” is defined and measured. Porter [1] quite rightly points out that what are used as measures of quality may have no bearing whatsoever to a “customer”-centered perspective on quality.

In the present health care delivery structure in the United States, several stakeholders have treatment decision responsibility and agency. These include, but are not necessarily limited to, the patient, the physician, the health care facility (including, but not exclusively, hospitals), the third-party payer (private insurance companies or public agencies), and various government entities. All of these stakeholders will have specific interests and motivations, which, most likely, will not be aligned with a global “societal” perspective. This, invariably, leads to conflicts in judgment of the value of specific treatments. A prior collaboration has previously shown that this stakeholder-perspective approach can provide insight into value analysis of robotic pancreas and liver surgery [2]. The purpose of this study is to model a framework to quantify the factors that may go into treatment decisions by patients, surgeons, hospitals, and private third-party payers. We will use the example of surgical treatment decisions for gastroesophageal reflux disease (GERD) to illustrate this concept.

## Materials and methods

Development of stakeholder value equations

We developed value propositions from the patient, surgeon, hospital, and private payer perspective. We acknowledge that these are not the only stakeholders in treatment decisions. However, we will not address other stakeholders, such as family members, employers, or local, state, or federal governments.

Patient Perspective

It goes without saying that the patient wants what is “best” for him or herself. However, what is that exactly and at what cost? These factors were determined by patient input over many years of a surgical practice.

Survival, measured in years (T): Any intervention can lengthen, shorten, or have no impact on a patient’s longevity.

Change in quality of life (Q), measured by a quality multiplier (QM): Most patients seek medical care because of some symptom or constellation of symptoms and afflictions that make their quality of life worse.

Satisfaction with care rendered (S): The patient’s experience with the health care provider, facility, or other actors (e.g., the insurance company) in his or her care may affect how they perceive the value of their care [3-5].

Out-of-pocket costs (C): Depending on the insurance coverage, many patients will be responsible for some portion of the costs of care.

Income loss (L): Some patients will lose income while they recover from an intervention, while others will not.

Health insurance premium (P): Some patients will have to pay some or all of the health insurance premium. For the purposes of this study, we assumed that the anti-reflux surgery will be the only episode of care.

Therefore, we determine the value for a patient as follows:

Value = TQS/(C+L+P), measured in quality-adjusted life years (QALYs)/$

Physician Perspective

The physician’s motivations, beyond having a good patient outcome, are several.

Income generated by the health care episode (I): A practicing physician, especially in a private practice, is required to earn an income. We assumed a fee-for-service setting, with each encounter will generate a fee of which some or all will be collected.

Reputation (R): A physician's reputation may affect referrals and income [6]. By rendering care for a particular spectrum of conditions, the surgeon will enhance (or diminish) his or her reputation.

Knowledge gained (K): With each patient encounter, the physician will increase his or her experience. Therefore, there is value in the form of knowledge gain during patient encounters [7].

Job satisfaction (S): Physicians choose particular disease processes and procedures upon which to practice, increasing job satisfaction [8,9].

Practice cost (C): The costs of this practice environment must be paid by the physician [10-12].

Opportunity loss (L): There may be opportunity loss when a physician cares for one type of patient rather than another [13,14].

Effort expended (E): Each patient encounter will require effort by the physician. In the setting of a global fee, capitation, or bundle-services reimbursement model, this will include the operation and all postoperative care for at least 90 days.

Therefore, we quantify physician value as follows:

Value = [R(I+K+S)-(C+L)]/E, measured in $/hour.

Hospital Perspective

The hospital also has several interests.

Income generated (I): We assumed with each operation, the hospital generates income, usually through facility fees in a fee-for-service model.

Reputation (R): Reputation is of great importance to the hospital as physicians and patients will choose to utilize the hospital based on its reputation and, therefore, can increase or decrease income because of its reputation [15].

Knowledge gain (K): The hospital, through its employees, will gain knowledge with patients with particular diseases and issues.

Cost to render care (C): The hospital has numerous fixed and variable costs that are related to rendering care to patients.

Opportunity loss (L): The potential increased in profit by care of another patient is an opportunity loss for the hospital [16].

Episode of care (E): The hospital will generate revenue, and change its reputation and knowledge as well as incur costs with each patient encounter.

Therefore, we determine that the value for the hospital with each patient encounter is as follows:

Value = [R(I+K)-(C+L)]/E, measured in $/episode.

Private Payer Perspective

From the private payer perspective, their goal is to maximize profits. Therefore, their interest is simply.

Income generated through premiums (I): This is the amount of revenue generated through the patient premium.

Cost due to pay-outs (C): This is the amount paid for an episode of care to the physician and hospital, as well as to others.

Time period covered by the premium (t): As contracts with an insurer generally cover a set period of time, usually one year, the insurer will profitability based on the fiscal year.

Therefore, from the private payers perspective, the value is as follows:

Value = (I-C)/t, measured in $/year.

Quantification of factors in GERD treatment decisions

We will use GERD surgical treatment decisions to illustrate these value calculations. We reviewed the literature of costs and outcomes of each of four GERD surgeries for each stakeholder|: patient, surgeon, hospital, and private payor. The four GERD operations are laparoscopic Nissen fundoplication (LNF), laparoscopic Toupet fundoplication (LTF), total incisionless fundoplication (TIF) using the EsophyX® device (EndoGastric Solutions, Inc., Redmond, WA), and laparoscopic magnetic sphincter augmentation (MSA) using the Linx® device (Johnson & Johnson, New Brunswick, NJ).

Operation cost: The costs of the operations for patients were the median costs obtained from the literature [17,18].

Income: Income generated for surgeons and hospitals was obtained from established Center for Medicare and Medicaid Services rates for laparoscopic esophagogastric fundoplasty [19,20].

Hospital costs: Hospital costs for these operations were obtained from the literature [18,21,22].

Third-party payer costs: Third-party payer costs were based on the combined physician and hospital reimbursement [19,20].

Surgeon’s effort: Surgeon’s effort expended is measured in hours [23,24].

Patient survival: We assumed the perspective of a healthy 40-year-old with the average United States life expectancy of 78 years, that is, a 38-year survival [25].

Patient income loss: We assumed a patient’s income loss of $3,625, based on the U.S. median household income of $62,843 per year [26], and 3 weeks estimated time lost from work.

Patient satisfaction and quality of life: Patient satisfaction with the clinical outcome and change in quality of life were estimated from patient-reported outcomes [27-31]. For the purposes of calculating the postoperative Q, we assumed that patients who are satisfied with their postoperative outcome have a QM of 1. We defined the procedure satisfaction rate as Qsatisfied and the procedure dissatisfaction rate as Qdissatisfied. We assumed that the QM of the dissatisfied patients will be based on the quality of life level of patients dissatisfied with their symptomatic outcome.

Calculating Q and S is as follows. We have shown that patients satisfied with their treatment (either medical or surgical), had on average 43% higher score than dissatisfied patients [32]. Therefore, the Q for each treatment is rate of patients satisfied with their treatment plus this rate multiplied by 0.57, i.e., Q=[(Qsatisfied)(1)+(Qdissatisfied)(0.57)]. S, we will use an across-the-board estimate of 0.98 for all procedures.

Patient premiums and out-of-pocket costs: The average employer-based costs for health insurance for a family is $20,576/year, with $6,015/year paid by the employee and the remainder paid by the employer [33], with the average annual deductible of $1,655/year [33]. We will assume that all of the deductible will be used for this episode of care.

Surgeon practice cost and opportunity loss: We used one estimate of practice expenses at $6,000/month [34]. We prorated the practice expense cost based on a 50-hour work week for a surgeon (minus two weeks’ vacation). This figure multiplied by the effort time will be used as the surgeon’s practice cost (C). The opportunity loss (L) is quite variable depending on the practice, we elected to keep L at 0 under the assumption that the surgeon will have no more lucrative alternative.

Hospital opportunity loss: We elected to keep L for the hospital at 0 under the assumption that the hospital will not have another more lucrative operation to place into that operating room.

Surgeon and hospital reputation, knowledge gained, surgeon job satisfaction: We somewhat set the reputation multiplier (R) at 1.1. We set the value of knowledge gained at $50/episode for the surgeon and $100/episode for the hospital and kept the same values for all procedures. We set the job satisfaction value at $100/episode for the surgeon and kept the same value for all procedures.

Private insurance income: Private payer’s average income generated through premiums is $20,576,49, which includes both portions from the employee and the employer. This premium will cover the patient for one year.

## Results

Stakeholder valuation estimates

Patient Valuation

Table 1 presents the data used for the value calculations from the patient’s perspective. Out-of-pocket costs will be considered the insurance deductible. Due to lack of data and for simplicity, we assumed the same income loss for each treatment. We also assumed the same insurance premium. For the Nissen fundoplication, the patient value is 2.99 QALYs/$1,000 spent. For the Toupet fundoplication, it is 2.87 QALYs/$1,000 spent. For the TIF procedure, it is 2.88 QALYs/$1,000 spent. Finally, for the Linx procedure, it is 2.89 QALYs/$1,000 spent.

**Table 1:** Patient Perspective Value Data Calculations SM, satisfaction multipler; TIF, total incisionless fundoplication; MSA, magnetic sphincter augmentation

Type of surgery	Patient’s out-of-pocket cost of care ($)	Change in survival (years)	Change in quality of life (quality multiplier)	Satisfaction with care (SM)	Income lost ($)	Premium or taxes paid for healthcare ($)
Nissen	$1,655	38	0.908	0.98	$3,625	$6,015
Toupet	$1,655	38	0.871	0.98	$3,625	$6,015
TIF	$1,655	38	0.874	0.98	$3,625	$6,015
MSA	$1,655	38	0.878	0.98	$3,625	$6,015

Physician Valuation

Table 2 shows the data used for the physician calculations. Reimbursement is based on the Medicare fee schedule for these procedures. The effort includes a 30-minute preoperative counseling visit, operative time, 30-minute in-hospital visit and discharge, and two 15-minute postoperative visit, all within the global fee. For simplicity, as there are no data, the reputation factor, job satisfaction value, and knowledge gained values were arbitrarily set and equal among the treatment options as not to introduce undocumented variables. With this, the value is $752.20/hour for a Nissen fundoplication, $529.06/hour for a Toupet fundoplication, $286.01/hour for TIF, and $346.96/hour for Linx.

**Table 2:** Surgeon Perspective Value Data Calculations RM, reputation multipler; TIF, total incisionless fundoplication; MSA, magnetic sphincter augmentation

Type of surgery	Income generated ($)	Surgeon’s effort expended (hours)	Change in reputation (RM)	Knowledge gained ($)	Job satisfaction ($)	Practice costs ($)	Opportunity loss ($)
Nissen	1,625	2.5	1.1	50	100	72.00	0
Toupet	1,625	3.5	1.1	50	100	100.80	0
TIF	451	2.1	1.1	50	100	60.48	0
MSA	704	2.5	1.1	50	100	72.00	0

Hospital Valuation

Table 3 shows the data used from the hospital calculations. We used equivalent income generated for each procedure, as this is based on standard facility fees. As with the surgeon, the reputation factor was kept constant. Therefore, the value for a Nissen fundoplication was $3,446/episode, $1,777/episode for a Toupet fundoplication, $1,277/episode for TIF, and $1,677/episode for Linx.

**Table 3:** Hospital Perspective Value Data Calculations RM, reputation multipler

Type of surgery	Change in reputation (RM)	Income generated ($)	Knowledge gained ($)	Cost to render care (direct and indirect) ($)	Opportunity loss ($)	Episode of care ($/episode)
Nissen	1.1	6,970	100	4,331	0	1
Toupet	1.1	6,970	100	6,000	0	1
Esophyx	1.1	6,970	100	6,500	0	1
Linx	1.1	6,970	100	6,100	0	1

Private Third-Party Payer

Table 4 shows the data for the private third-party payer calculations. The insurance company will still be profitable by $12,162/year for Nissen and Toupet fundoplications, $13,336/year for TIF, and $13,083/year for MSA.

**Table 4:** Private Third-Party Payer Value Data Calculation

Type of surgery	Income generated through premiums	Costs due to payouts to providers ($)	Time period covered by premium ($/year)
Nissen	20,757	8,595	1
Toupet	20,757	8,595	1
Esophyx	20,757	7,421	1
Linx	20,757	7,674	1

## Discussion

What we hoped to accomplish with this analysis was to develop a first iteration model quantifying the value that the four principal stakeholders will perceive in the decision to proceed with an anti-reflux operation. Although we acknowledge the constraints in obtaining precise data for each of the factors in the formulas used, our aim was to stimulate a more quantified way of assessing treatment value for individual stakeholders. We have tried to identify and quantify most of the motivations for each stakeholder. We acknowledge that motivations we have used may not account for all of the motivations in each individual decision (e.g., family influence for patients, public relation considerations for third-party payers and hospitals, ethical considerations for surgeons), but we feel that we have accounted for the ones most quantifiable. We believe that the most important finding, although not surprising and obvious to even the most casual observer, is that the “units of measure” are different for each stakeholder and therefore an “apples to apples” comparison among stakeholders is not possible. Moreover, these different units of measure may explain the conflicts among stakeholders.

We believe that we can take for granted that patients want to improve their health, extend their life, and improve their quality of life when seeking healthcare. Beyond that, several studies have attempted to address patient treatment decision-making in a variety of settings [35-39]. Out-of-pocket costs can affect a patient's treatment decision. Specifically, with anti-reflux surgery, many insurance companies consider the EsophyX device and Linx device “experimental” and will not pay for these procedures. This can be a potential out-of-pocket cost to a patient and affect their decision to proceed with these procedures. Lastly, satisfaction with the process of care is certainly believed to affect patient choice [40]. Our formula attempts to take into account all of these factors into a single value.

There are several motivations for physicians in choosing a specialty and types of patients and interventions they will seek to see and do [8,41-43]. All of these motivations will eventually have to be integrated into the business of a medical practice. After income generation, motivations with respect to enhancing reputation, job satisfaction, and knowledge expansion become more important, although much more nebulous to quantify.

A hospital is a business that needs to generate enough revenue to meet its expenses. They are engaging in competition with surrounding hospitals for patients and physicians [15]. Elective admissions and procedures generate a substantial portion of overall revenue [44,45]. Although difficult to quantify, reputation and knowledge gained are of value after income generation.

By necessity, many of the values used in the calculations are estimates and/or arbitrary. Therefore, the exact values as determined will vary depending on the actual values for a particular individual patient, surgeon, hospital, and third-party payer. We also assumed the “best-case scenario” of a patient undergoing the procedure without complications and life-long relief of symptoms. It is clear that for all the anti-reflux operations assessed here, there is a certain number of patients who will have recurrent symptoms or new adverse symptoms, such as dysphagia, who will require additional intervention, thereby reducing the value of the initial anti-reflux operation. It is therefore obvious that in treatment options that may not convey a life-long consistent improvement, the valuation formulas used will need modification. We also did not take into account the occurrence of postoperative complications, which will also change the value for all stakeholders. Modifications to the formulas will be required to account for these events. We also used only the deductible as the patient’s out-of-pocket cost. Clearly, the patient and family may have additional out-of-pocket costs, such as travel, or the deductible may have been met prior to the operation so as not to factor into the valuation for this episode of care. Income loss and premiums will also vary. Similarly, for the surgeon and hospital, income generated will depend on contracts with payers and payer mix, whereas we used Medicare reimbursement rates. How a surgeon would value reputation enhancement, knowledge gained, and job satisfaction, and, similarly, how a hospital would value reputation enhancement and knowledge gained is completely unknown. Lastly, income and costs as relating to third-party payers will vary depending on contracts and additional pay-outs related to an insuree’s medical utilization. These payers may also have other considerations, kept relatively opaque from the public, which also determine how they would value treatments. An example would be, although this is pure speculation, that refusing coverage for new devices or procedures, although individually more cost-effective for the payer, sets a precedent requiring the payer to pay for other more expensive treatments.

 Future directions for this research is to survey the stakeholders on whether the factors and values as presented correspond to actual decision-making. Also, a survey of stakeholders for the actual values of actual outcomes, including less than ideal outcomes, would better determine true valuation.

## Conclusions

In conclusion, we presented a model of stakeholder value for treatment decisions. Using the example of surgical management of GERD, LNF has the highest value for each stakeholder. We acknowledge that different values used in the formulas will lead to different valuations by the stakeholders.
